# Cell Type Mediated Resistance of Vesicular Stomatitis Virus and Sendai Virus to Ribavirin

**DOI:** 10.1371/journal.pone.0011265

**Published:** 2010-06-22

**Authors:** Nirav R. Shah, Amanda Sunderland, Valery Z. Grdzelishvili

**Affiliations:** Department of Biology, University of North Carolina at Charlotte, Charlotte, North Carolina, United States of America; Erasmus Medical Center, Netherlands

## Abstract

Ribavirin (RBV) is a synthetic nucleoside analog with broad spectrum antiviral activity. Although RBV is approved for the treatment of hepatitis C virus, respiratory syncytial virus, and Lassa fever virus infections, its mechanism of action and therapeutic efficacy remains highly controversial. Recent reports show that the development of cell-based resistance after continuous RBV treatment via decreased RBV uptake can greatly limit its efficacy. Here, we examined whether certain cell types are naturally resistant to RBV even without prior drug exposure. Seven different cell lines from various host species were compared for RBV antiviral activity against two nonsegmented negative-strand RNA viruses, vesicular stomatitis virus (VSV, a rhabdovirus) and Sendai virus (SeV, a paramyxovirus). Our results show striking differences between cell types in their response to RBV, ranging from virtually no antiviral effect to very effective inhibition of viral replication. Despite differences in viral replication kinetics for VSV and SeV in the seven cell lines, the observed pattern of RBV resistance was very similar for both viruses, suggesting that cellular rather than viral determinants play a major role in this resistance. While none of the tested cell lines was defective in RBV uptake, dramatic variations were observed in the long-term accumulation of RBV in different cell types, and it correlated with the antiviral efficacy of RBV. While addition of guanosine neutralized RBV only in cells already highly resistant to RBV, actinomycin D almost completely reversed the RBV effect (but not uptake) in all cell lines. Together, our data suggest that RBV may inhibit the same virus via different mechanisms in different cell types depending on the intracellular RBV metabolism. Our results strongly point out the importance of using multiple cell lines of different origin when antiviral efficacy and potency are examined for new as well as established drugs in vitro.

## Introduction

Ribavirin (RBV, also known as virazole), 1-*ß*-D-ribofuranosyl-1,2,4-triazole-3-carboxamide, is the first synthetic, broad-spectrum antiviral nucleoside analog [1], which has been shown to exhibit antiviral activity against many RNA and DNA viruses both in vitro and in vivo [2], [3], [4], [5]. RBV was originally approved for the treatment of respiratory syncytial virus (RSV) infection in children, and today is also used to treat Lassa fever and, most importantly, hepatitis C virus (HCV) infections of humans [4]. While RBV alone has little or no effect on viral replication in HCV patients [6], it dramatically improves long-term antiviral response in many treated patients when used in combination with interferon (IFN) [3], [4]. The mechanism of synergy between RBV and IFN [7], [8], which is critical for successful anti-HCV therapy, remains unclear [4].

Despite these successes with RBV/IFN combination therapy resulting in a so called sustained virological response (SVR, no detectable plasma HCV RNA during treatment and for at least 6 months after therapy) or end-of-treatment response (ETR, HCV RNA is undetectable when therapy is terminated), a large portion of patients are “non-responders” (detectable HCV RNA throughout the treatment period). The mechanism of non-response to RBV/IFN treatment is very controversial and, unfortunately, no alternative therapies available for non-responders.

The understanding of RBV treatment failures is complicated by an unclear mechanism of RBV action, partly due to its apparent pleiotropic nature [3], [4]. Upon uptake, RBV is metabolized in vivo through 5′-phosphorylation by cellular kinases into ribavirin mono- (RMP), di- (RDP) and triphosphate (RTP) [9], [10], [11]. Six distinct mechanisms (which may work together) have been proposed for antiviral action of RBV against different viruses [2], [3], [4], [5]: i) inhibition of the host enzyme inosine monophosphate dehydrogenase (IMPDH) essential for the *de novo* synthesis of GTP; ii) direct interaction of phosphorylated RBV with and inhibition of viral RNA polymerase, iii) RNA chain termination as a result of incorporation of RTP (GTP analog) into replicating RNA strands by viral RNA polymerases; iv) “error catastrophe” as a result of RTP incorporation into the viral genome paired with cytidine and uridine as a substitute for guanine and/or adenine, resulting in so called “lethal mutagenesis”, a meltdown of genetic information; v) inhibition of mRNA capping; and vi) immunomodulation of antiviral cellular responses such as the ability to induce a Th2 to Th1 shift in the immune response.

Previous studies in search of explanations for RBV treatment failures were largely focused on the role of viral determinants of RBV resistance [5], [6], as any antiviral mechanism of RBV via direct interactions with the viral RNA polymerase can hypothetically be overcome by mutations in the viral RNA polymerase. Such an escape via a single mutation in the RNA-dependent RNA polymerase has been shown to confer resistance to RBV via increased polymerase fidelity in poliovirus [5], [12] and foot-and-mouth disease virus [13], [14].

While drug resistant viral mutants may explain at least some failures with RBV treatments, recent reports propose that cell-based resistance to RBV could be an important factor explaining the low antiviral activity of RBV in at least some experimental and clinical systems [6]. For example, Pfeiffer and Kirkegaard provided in vitro evidence that resistance of infected cells to RBV can be conferred not only via mutations in the viral genome (“virus-based resistance”) but also through changes in the RBV treated cells (“cell-based resistance”) [12], [15]. A recent study by Ibarra and Pfeiffer [16] shows that the development of cell-based resistance to RBV treatment via decreased RBV uptake can greatly limit RBV antiviral activity.

To examine whether certain cell types are naturally resistant to RBV even without prior drug exposure, we selected seven different cell lines from various hosts and compared them for the antiviral activities of RBV against two nonsegmented negative-strand RNA viruses (order *Mononegavirales*), vesicular stomatitis virus (VSV, family *Rhabdoviridae*) and Sendai virus (SeV, family *Paramyxoviridae*), which were previously shown to be highly sensitive to RBV treatment [17], [18], [19], [20], [21]. Our results show dramatic cell-type dependent differences in the antiviral activities of RBV, ranging from virtually no effect to very effective inhibition of viral replication, indicating that some cell types are naturally resistant to RBV treatment even without prior exposure to this drug. The data presented in this study shed light on the mechanisms of the RBV activity against VSV and SeV, and may explain at least some of the reported failures with RBV treatments.

## Materials and Methods

### Cell lines and viruses

The following seven cell lines were used in this study: Syrian golden hamster kidney fibroblast cells (BHK21, ATCC# CCL-10); human cervical adenocarcinoma cells (HeLA, ATCC# CCL-2); human epithelial lung carcinoma cells (A549, ATCC# CCL-185), mouse mammary gland adenocarcinoma cells (4T1, ATCC# CRL-2539), human epidermal carcinoma cells (HEp2, ATCC# CCL-23); and African green monkey kidney cells (Vero, ATCC# CCL-81). In addition, we used BSRT7 cells which are derived from BHK21, constitutively express bacteriophage T7 polymerase and described by Buchholz et al. [22]. Monolayer cultures of these cell lines were maintained in Minimum Essential Medium (Eagle's MEM, Cellgro) or Dulbecco's modified Eagle's medium (DMEM, Cellgro) supplemented with 9% fetal bovine serum (FBS, Gibco) in a 5% CO_2_ atmosphere at 37°C. VSV-GFP is a recombinant wild type (wt) VSV (Indiana serotype) encoding GFP as an extra gene between the G and L genes [23], kindly provided by Dr. Asit K. Pattnaik (University of Nebraska). Recombinant SeV-GFP (Fushimi strain) encoding GFP upstream of the NP gene [24] was kindly provided by Dr. Wolfgang J. Neubert (Max-Planck-Institute of Biochemistry, Germany). To grow VSV-GFP or SeV-GFP, BHK21 or Vero cells, respectively, were infected with viruses at a multiplicity of infection (MOI) of 0.05 CIU (cell infectious units) per cell in MegaVir HyQSFM4 serum-free medium (SFM, Hyclone) and incubated for 24–48 h at 34°C. This temperature (34°C) was chosen as it supported optimal replication of both viruses in the seven cell lines (data not shown) and all virus infections presented in this study were conducted at 34°C. SeV-GFP was grown without acetylated trypsin in the medium as it has the wt monobasic trypsin-dependent cleavage site in the F protein mutated to an oligobasic cleavage site, allowing F activation in any cell type through an ubiquitous furin-like protease [24].

### Inhibitors

RBV was purchased from MP Biomedicals (cat. no. 196066); guanosine (cat. no. 101907) and actinomycin D (ActD) (cat. no. 10465805) from MP Biomedicals; and *S*-(4-Nitrobenzyl)-6-thioinosine (NBMPR, also known as NBTI, cat. no. N2255) from Sigma-Aldrich. Stock solution of RBV (0.1 M) was made in H_2_O, while ActD (2 mg/ml) was dissolved in 100% ethyl alcohol, and guanosine (20 mM) and NBMPR (16.8 mM) in DMSO.

### Virus infections in the presence of inhibitors

Most experiments were conducted using 24-well tissue culture plates and nearly 100% confluent cells treated with drugs in SFM (or mock-treated with SFM) and infected with VSV-GFP or SeV-GFP (or mock-infected with SFM) at MOI of 3 CIU/cell. The MOI for each virus/cell type combination was calculated by infecting each cell line with VSV-GFP or SeV-GFP serial dilutions in SFM and counting infectious foci with the aid of fluorescence microscopy. RBV was added to the cells at 24 h before infection. After absorption of virus for 1 h in the absence of drugs (to rule out an interference of drugs with virus attachment/entry), SFM containing unabsorbed virus was removed, cells were washed three times with phosphate-buffered saline (PBS), and 300 µl/well of SFM with the same concentrations of drugs as in the pretreatment was added to each well. The fluorescence and bright field photographs of cells at 10× magnification were captured 24 h post infection (p.i.) or 48 h p.i. using an Olympus DP70 digital camera mounted on an Olympus IX71 inverted fluorescent microscope and Olympus DP Controller software. To examine effect of RBV on virus production, SFM containing infectious particles was collected 24 or 48 h p.i., and viral titrations were performed in 96-well plate format by infecting BHK21 (for VSV) or Vero cells (for SeV) with serial virus dilutions. For SeV titration, cells were overlaid with 100 µl SFM containing 1.2% Avicel RC-581 (FMC BioPolymer, Philadelphia, PA) as previously described [25], while a 0.5× SFM/1% bactoagar mixture was used to overlay VSV-infected cells.

The effect of the exogenously added guanosine on VSV and SeV replication in the presence or absence of RBV was examined using confluent monolayers of cells in 96-well tissue culture plates (performed three times, done in triplicates). Cells were infected with either VSV-GFP or SeV-GFP (or mock-infected with SFM) at MOI of 3 CIU/cell. After 1 h p.i., virus was removed and cells were washed with PBS, and mock-treated or treated with the SFM containing 500 µM RBV or 50 µM guanosine, or RBV together with guanosine. Guanosine was dissolved in DMSO and the final concentration of DMSO in the media added to all wells was 0.25%. The intensity of fluorescent signal at 18 h p.i for VSV and 24 h p.i for SeV was quantified using a Fluorescence Multi-Well Plate Reader CytoFluor 4000 (PerSeptive Biosystems, Inc., Framingham, MA) with the standard in built Cyto Fluor filter set (excitation wavelength at 485 and emission wavelength at 530 nm). Values were corrected for background fluorescence by subtracting the values of uninfected cells from the value of each infected well.

### Plaque reduction assay to determine RBV inhibitory concentrations

To estimate the 50% and 90% inhibitory concentrations (IC_50_ and IC_90_) for RBV, antiviral screening was conducted by means of a plaque reduction assay using 24-well tissue culture plates. Cells were infected with VSV-GFP or SeV-GFP in SFM (or mock-infected with SFM) at an MOI producing about 100 virus plaques per well on each cell line in the absence of RBV. After absorption of virus for 1 h without RBV (to rule out an interference with virus attachment/entry), SFM containing unabsorbed virus was removed, cells were washed three times with phosphate-buffered saline (PBS), and overlaid with 200 µl/well of SFM containing 1.2% Avicel RC-581 and increasing concentrations of RBV. Cells were then incubated for 24 h (VSV) or 48 h (SeV). Plaques were counted with the aid of fluorescence and bright field microscopy, and the 50% (IC_50_) and 90% (IC_90_) inhibitory concentrations were calculated. Initial experiments were done using 0, 200, 500 or 1000 µM of RBV as it was done for virus infections at MOI 3 to determine the range of RBV activity for each virus/cell line combination. After that, all plaque reduction experiments were conducted using different ranges of RBV concentrations to more precisely determine the IC_50_ and IC_90_ values. Each of these experiments was performed at least twice (done in duplicates) and plaque numbers represent the mean ± standard deviation of the mean.

### Virus growth analysis

The relative efficiency of the initiation of infection by VSV-GFP and SeV-GFP was measured by titrating viruses on the seven cell lines to determine the number of viral particles successfully initiating infection in a given cell line. For one-step growth kinetics analysis, confluent cell monolayers in 24-well plates were infected in parallel at an MOI of 3 CIU/cell. At 1 h p.i., infection medium was aspirated, cells were washed three times with PBS (to minimize carryover of virions), and 300 µl of fresh SFM was added to each well. SFM from each well was collected at the specified time intervals, flash frozen at −80°C, and analyzed by titration as described above.

### RBV uptake assay

Cell monolayers were prepared exactly as for virus infections using 12- or 24-well tissue culture plates. The [^3^H]RBV uptake experiments were conducted essentially as in [16] but with some modifications. Cells were plated the day prior to generate about 90% confluence on the day of the experiment. For RBV uptake in the presence or absence of NBMPR (15 or 100 µM), cells (in triplicates) on 24-well plates were pretreated with this nucleoside transporter inhibitor in DMSO (or with DMSO alone) for 15 minutes. Cells were then washed with PBS and treated with 100 µl of SFM (same medium used for infections but without virus) containing 50 µM RBV 1% of which was [^3^H]RBV (ViTRax, Placentia, CA, cat. no. VT193, specific activity 5 Ci/mmol) for 15 minutes in a 5% CO_2_ atmosphere at 37°C. For the long-term accumulation of RBV, cells (in triplicates) on 12-well plates were washed with PBS and treated with 275 µl of SFM (same medium used for infections but without virus) containing the same concentration of RBV/[^3^H]RBV (in the absence of NBMPR) as above but incubated for 1 h, 16 h or 24 h. To measure intracellular [^3^H]RBV, cells were then placed on ice for 5 minutes (to stop an uptake) and washed 3 times with cold PBS. The cells were then trypsinized, pelleted at 200×g for 4 minutes and cell pellets were frozen at −80°C. Nucleotide pool isolation was conducted as described in [26]. Specifically, tubes with frozen cell pellets were placed on ice and 75 µl of 1.3 N cold formic acid was added to each pellet, cell pellets were resuspended in formic acid and incubated for 1 h (tubes were vortexed every 15 minutes) on ice. After 1 h extraction period, the formic acid suspension was centrifuged at 17,000×g, and the supernatant extracts (75 µl) were transferred to new tubes and quantified (15 µl) by scintillation counting for the intracellular [^3^H] accumulation. Cell numbers (from separate plates) were counted by two separate methods. First, cells were trypsinized and cell number was determined using a hemocytometer. Cell numbers were independently confirmed by staining monolayers (from a separate plate) with blue-fluorescent Hoechst 33342 dye (Invitrogen), which selectively stains nuclei. At least 5 random fields were photographed using a fluorescence microscope and DAPI filter and nuclei were then counted. Uptake values were determined by dividing the counts per minute (CPM) by number of cells (CPM/cell) in a 24-well plate. For RBV uptake in the presence of ActD, cells were pretreated with 5 µg/ml ActD for 2 h, media was aspirated (without cell washing), and then RBV uptake assay was conducted as described above.

### Cell viability assays

Cellular toxicity of RBV was determined using about 80% confluent cells treated with increasing RBV concentrations (0, 200, 500 or 1000 µM) at 37°C and 5% CO_2_ for 24 h. After 24 h, all cells reached 100% confluence and were analyzed by the following three assays: i) MTT (Biotium, cat. no. 30006, 96-well plate format) cell viability assay; ii) CellTiter-Glo luminescent cell viability assay (Promega cat. no. G7570, 96-well plate format); and iii) cell counting using trypan blue dye exclusion as an indicator of live cells (24-well plate format). MTT assay was conducted according to the manufacturer's (Biotium) protocol. Briefly, after 24 h incubation with RBV, 10 µl of MTT solution was added to each well and cells were incubated for 4 h at 37°C. Media was then removed and 200 µl of DMSO added to each well. OD values were measured using a Multiskan Ascent Microplate Photometer (Thermo Fisher Scientific) at a test wavelength of 570 nm and reference wavelength of 630 nm to determine the OD_570_–OD_630_ signal. CellTiter-Glo assay was conducted according to the manufacturer's (Promega) protocol and using 96-well white opaque culture plates (PerkinElmer, cat. no. 6005680). After 24 h incubation with RBV, 100 µl of CellTiter-Glo reagent was added to each well, plates were mixed for 2 minutes on orbital shaker to induce cell lysis, and incubated for 10 minutes to stabilize the luminescence signal. Luminescence was measured using Perkin Elmer TopCount NXT microplate luminescence counter. For trypan blue dye exclusion, 24-well plates were used. After 24 h incubation with RBV, cells were trypsinized and the number of viable cells was determined microscopically in a hemacytometer by trypan blue exclusion.

## Results

### Identification of RBV-resistant cell lines

**Figure 1:**
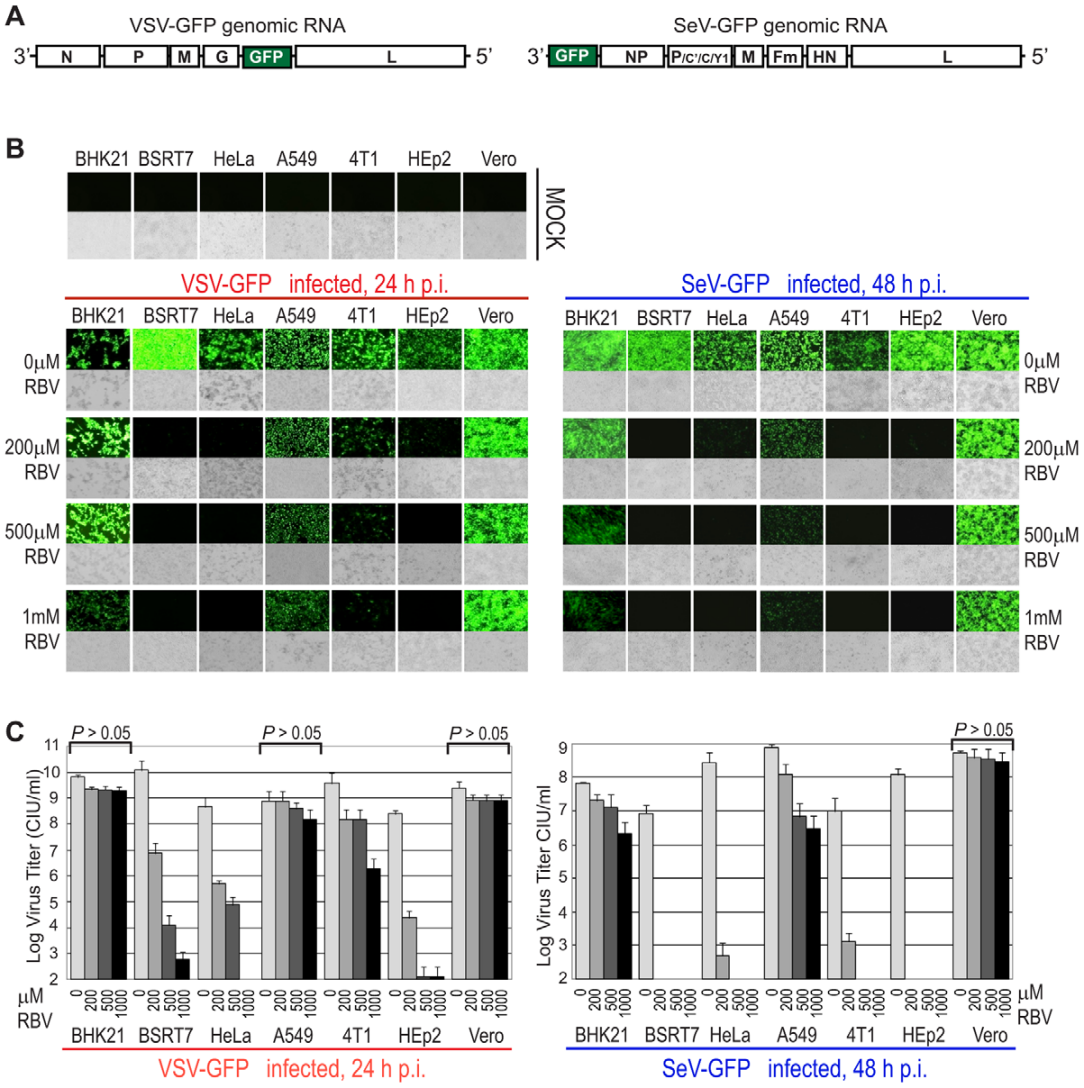
Effect of RBV on viral replication in seven cell lines. (A) The organization of the negative-sense RNA genomes of the recombinant viruses used in this study. (B) The panels show photographs of cells pretreated for 24 h with increasing concentrations of RBV as indicated (or mock-treated), infected with VSV-GFP (left) or SeV-GFP (right) at MOI 3 CIU/cell (or mock-infected, upper row), and then the same concentrations of drugs as in the pretreatment was added to each well after virus absorption. Fluorescence (upper panels) and light (lower panels) microscopy images were captured at 10× magnification. The photographs are typical representations of at least three independent experiments and an average field for each well is shown. (C) Media from the experiments described in B was collected at 24 h p.i for VSV (left) or at 48 h p.i for SeV (right) and virus titer was determined by standard plaque assay on BHK21 (for VSV) or Vero cells (for SeV). The data represent the mean ± standard deviation of two independent experiments (done in duplicates). Statistical analysis was done using one-way ANOVA with Tukey's post hoc test (GraphPad Prism 4, San Diego, CA). RBV treatments without significant decrease in viral titer at any tested RBV concentrations as compared to mock-treated cells (“0 µM RBV”) are indicated as *P*>0.05.

To determine whether “natural” (without pre-exposure to drug) resistance to RBV exists in some cell types, we selected seven commonly used cell lines (BHK21, BSRT7, HeLa, A549, 4T1, HEp2, and Vero) originated from various hosts and tissues, and compared them for the antiviral activity of RBV against VSV and SeV. To facilitate virus detection, we employed recombinant viruses containing an additionally inserted GFP gene (Fig. 1A). While such insertion results in a mild attenuation of VSV [23] and SeV [24], [27], both viruses replicate similarly to parental wt strains (data not shown) and, thus, serve as useful models for studying replication of wt viruses. Cells were treated with increasing concentrations of RBV added to the media 24 h before infection, and then infected with viruses at MOI of 3 CIU/cell with RBV treatment continued after virus absorption. The MOI for each cell line was calculated individually by titrating viruses on each of the seven cell lines as described in Materials and Methods and Table 1. Following RBV treatment and virus infection, pictures were taken 24 h post infection (p.i.) for VSV or 48 h p.i. for SeV using fluorescence and light microscopy. As shown in Figure 1B, GFP-associated fluorescence attributable to viral replication was readily detectable in all tested cells lines infected with VSV or SeV when no RBV was added to the media, indicating that all cell lines were susceptible to infection by these two viruses. Consistent with previous studies demonstrating antiviral activity of RBV against VSV, RBV effectively inhibited VSV in BSRT7, HeLa and HEp2 cells even at the lowest (200 µM) tested drug concentration (Fig. 1B). However, RBV had a surprisingly mild effect on the VSV-driven GFP expression in BHK21, Vero and A549 cells even when used at 1000 µM concentration with a somewhat intermediate effect in 4T1 cells (Fig. 1B). In general, RBV inhibited SeV replication to a greater degree than VSV with markedly stronger inhibition in 4T1 cells. However, Figure 1B clearly shows a similar pattern of RBV resistance in BHK21, Vero and A549 cells for VSV and SeV, suggesting that cellular rather than virus-specific factors determine the dramatic differences between tested cell lines in their response to RBV. A similar pattern was also observed when RBV was added to the medium 6 h (rather than 24 h) before or 1 h after infection (without RBV pretreatment), although in general RBV was more effective when longer pretreatments were conducted (data not shown). In addition, a similar pattern of RBV effect in the seven cell lines was observed when experiments were conducted at 37°C rather than at 34°C [34°C was chosen for experiments presented here as it supported optimal replication of both viruses in the seven cell lines (data not shown)] or with cells of various passage level (3 to 20 passages) or confluence (70%), demonstrating that the observed effect was not determined by the state of the cells (data not shown).

**Table 1:** Relative number of infectious virus particles added to different cell lines to achieve MOI 3 for each virus/cell combination.

	VSV	SeV
BHK21	3.0 CIU^BHK^/cell	7.5 CIU^HeLa^/cell
BSRT7	22.5 CIU^BHK^/cell	3.2 CIU^HeLa^/cell
HeLa	132.0 CIU^BHK^/cell	3.0 CIU^HeLa^/cell
A549	13.2 CIU^BHK^/cell	8.1 CIU^HeLa^/cell
4T1	227.0 CIU^BHK^/cell	37.8 CIU^HeLa^/cell
HEp2	227.0 CIU^BHK^/cell	4.8 CIU^HeLa^/cell
Vero	23.7 CIU^BHK^/cell	4.3 CIU^HeLa^/cell

Most experiments in this study were conducted using 24-well tissue culture plates and nearly 100% confluent cells treated with RBV in SFM (or mock-treated with SFM) and infected with VSV-GFP or SeV-GFP (or mock-infected with SFM) at MOI of 3 CIU/cell of the tested cell line. The MOI of 3 CIU/cell for each virus/cell type combination was calculated by infecting each cell line with serial dilutions of VSV-GFP or SeV-GFP virus stock in SFM and counting infectious foci with the aid of fluorescence microscopy (see Figure 7A). VSV CIU^BHK^ – number of cell infectious units (infectious particles) determined by titration of VSV-GFP virus stock on BHK21 cells. SeV CIU^HeLa^ - number of cell infectious units calculated by titration of SeV-GFP virus stock on HeLa cells.

To determine whether GFP levels correlated with the production of new infectious virus particles, the medium was collected and subjected to plaque assay on BHK21 (for VSV) or Vero (for SeV) cells. Virus titration analysis showed a clear correlation between GFP signal and the number of infectious virus particles produced in different cell lines under various treatment conditions (Fig. 1C).

**Figure 2:**
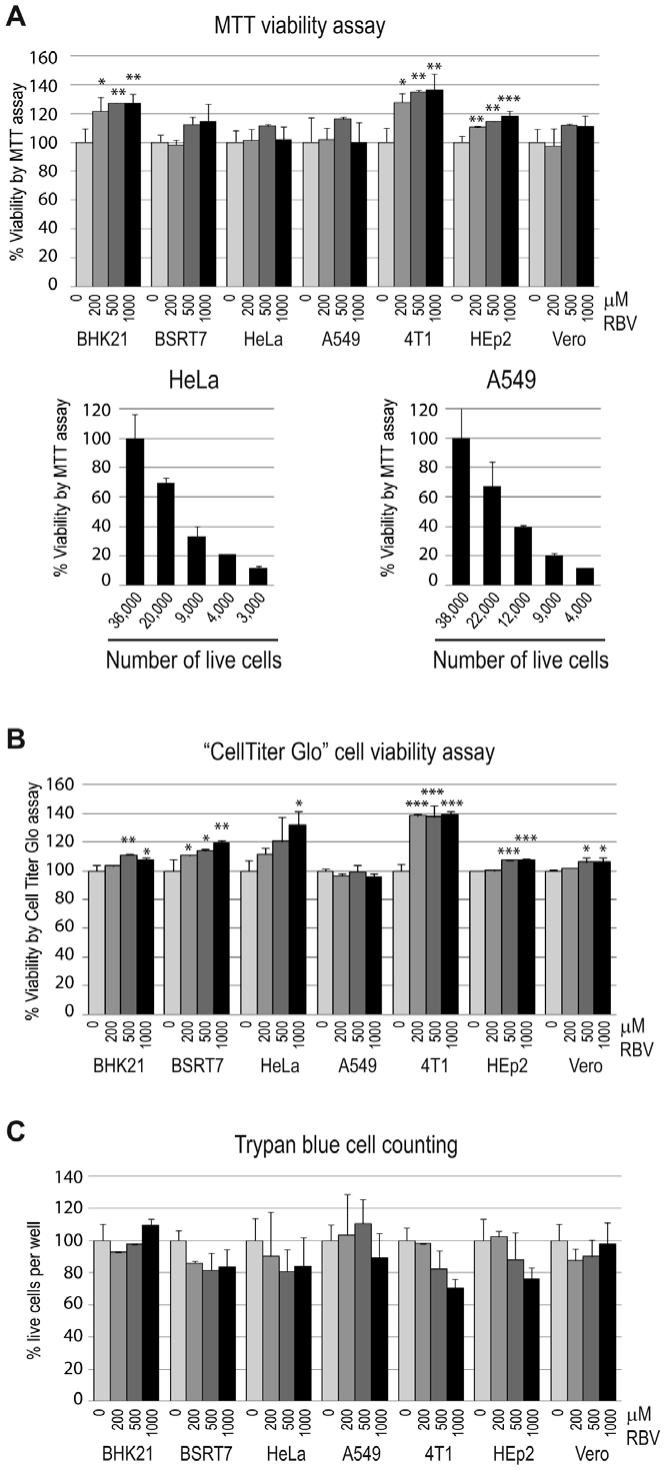
Effect of RBV on cell viability of seven cell lines. To determine the relative toxicity of increasing concentrations of RBV in different cell lines, 80%-confluent uninfected cells were treated with RBV for 24 h and tested for viability using MTT cell viability assay (A) or CellTiter-Glo Luminescent Cell Viability Assay (B) or by cell counting using trypan blue dye exclusion as an indicator of live cells (C) as described in Materials and Methods. To determine the sensitivity of the MTT assay, serial dilutions of A549 and HeLa cells were plated [lower left and right graphs in (A)], grown for 24 h, cells from separate wells were trypsinized and counted using a hemocytometer (36,000 cells for HeLa and 38,000 cells for A549 formed 100% confluent monolayers), and MTT assay was conducted as described in Materials and Methods. (A–C) The data (done in triplicate) represent the mean ± standard deviation and are expressed as a percentage of the untreated control. Statistical analysis was done using one-way ANOVA with Tukey's post hoc test (GraphPad Prism 4, San Diego, CA). ***P<0.001, **P<0.01, *P<0.05, as compared to mock-treated cells (indicated as 0 µM RBV).

Next, we examined a possibility that a higher sensitivity of VSV and SeV to RBV in 4T1, BSRT7, HeLa and HEp2 was due to the increased cellular toxicity of RBV in these cell lines, which could result in the decreased ability of these cells to efficiently support viral replication. To address this issue, we used three different assays to measure cell viability using cells prepared and RBV-treated the same way as for virus infections shown in Figure 1: i) colorimetric MTT assay based on the reduction of the yellow tetrazolium salt MTT to the insoluble purple formazan crystals, which are solubilized by the addition of a detergent in metabolically active cells (Fig. 2A); ii) luminescent “CellTiter-Glo” assay based on quantitation of the intracellular ATP content as an indicator of metabolically active cells (Fig. 2B); iii) live cell counting using trypan blue dye exclusion as an indicator cell membrane integrity in the live cells (Fig. 2C). Using these three different methods (as described in Materials and Methods), we showed that RBV treatment even at 1000 µM concentration did not produce any statistically significant decrease in cell viability in any of the tested cell lines under our experimental conditions (Fig. 2), indicating that the observed pattern of RBV antiviral activity was not due to the differential RBV cytotoxicity in the tested cell lines (Fig. 2). To prepare cells for these assays, 80% confluent cells were treated with RBV for 24 h (same conditions used for virus infections in Figure 1). After 24 h treatment, all tested cell lines reached 100% confluence suggesting that RBV did not produce any substantial cytotoxicity that would prevent cell growth. However, we recognize that the cell viability assays conducted on 100% confluent cells may not be sensitive enough to detect all adverse effects of RBV on the host cell. Nevertheless, the absence of significant drop in cell viability by 3 independent assays were in good agreement with the lack of visible differences between RBV treated and untreated cells using light microscopy (Figure 1B and data not shown).

**Figure 3:**
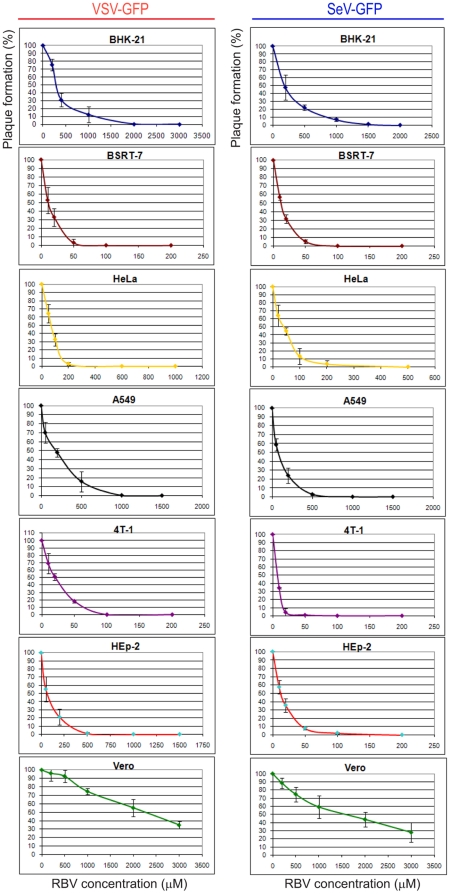
Plaque reduction assay to determine RBV inhibitory concentrations. Cell monolayers were infected with VSV-GFP or SeV-GFP (or mock-infected; 0 µM RBV) using virus dilutions producing about 100 virus (“100%”) on each cell line in the absence of RBV, overlaid with SFM containing 1.2% Avicel RC-581 and increasing concentrations of RBV (note that different RBV concentrations were used for each virus-cell type combination). Cells were then incubated for 24 h (VSV) or 48 h (SeV), and plaques were counted with the aid of fluorescence and bright field microscopy. “0%” indicates that no fluorescent infectious foci were detected. Each experiment was performed at least twice (done in duplicates) and data points represent the mean ± standard deviation.

All infection experiments described above were conducted at MOI of 3 CIU/cell to achieve one-step replication of viruses in all tested cell lines. We also conducted additional experiments with cells infected at MOI 0.2, 0.5, 1, 10 or 20 in the presence of increasing concentrations of RBV (same range as above) and observed a similar pattern of RBV resistance in Vero, BHK21 and A549, indicating that this effect was MOI independent (data not shown). To further confirm the MOI-independent character of RBV resistance in Vero, BHK21 and A549 cells, we conducted a plaque reduction assay in the presence of RBV, which also allowed us to calculate the 50% and 90% inhibitory concentrations (IC_50_ and IC_90_) of RBV for each virus/cell type combination, as described in Materials and Methods. As shown in Figure 3 and summarized in Table 2, the IC_50_ and IC_90_ values were in good agreement with our data using MOI 3 infections (Fig. 1). We find especially striking resistance of Vero cells to RBV with IC_50_ = 2250 µM for VSV and 1550 µM for SeV and IC_90_>3000 µM for both viruses. Compared to SeV, VSV was consistently more resistant to RBV in all tested cell lines, which might be associated with its markedly faster growth in all tested cell lines (addressed below). Nevertheless, the similar cell type dependent pattern of RBV resistance for VSV and SeV suggests that cellular determinants play a major role in RBV resistance.

**Table 2:** Antiviral activity (IC_50_ and IC_90_) of RBV against VSV and SeV in different cell types.

Cell type	IC_50_ (µM)	IC_90_ (µM)	IC_50_ (µM)	IC_90_ (µM)
	VSV-GFP		SeV-GFP	
BHK21	275	1100	190	850
BSRT7	10	40	16	40
HeLa	70	150	40	110
A549	190	610	90	320
4T1	20	60	10	18
HEp2	70	310	12	45
Vero	2250	>3000	1550	>3000

The 50% and 90% inhibitory concentrations (IC_50_ and IC_90_) for RBV were estimated by means of the plaque reduction (Fig. 2) as described in Materials and Methods. Data are expressed as mean without standard error of mean that, however, never exceeded 20% of the mean values. Note that an extremely poor potency of RBV against VSV and SeV in Vero did not allow it to reach IC_90_ even at 3000 µM RBV concentration (“>3000”).

### Analysis of RBV uptake in different cell lines

**Figure 4:**
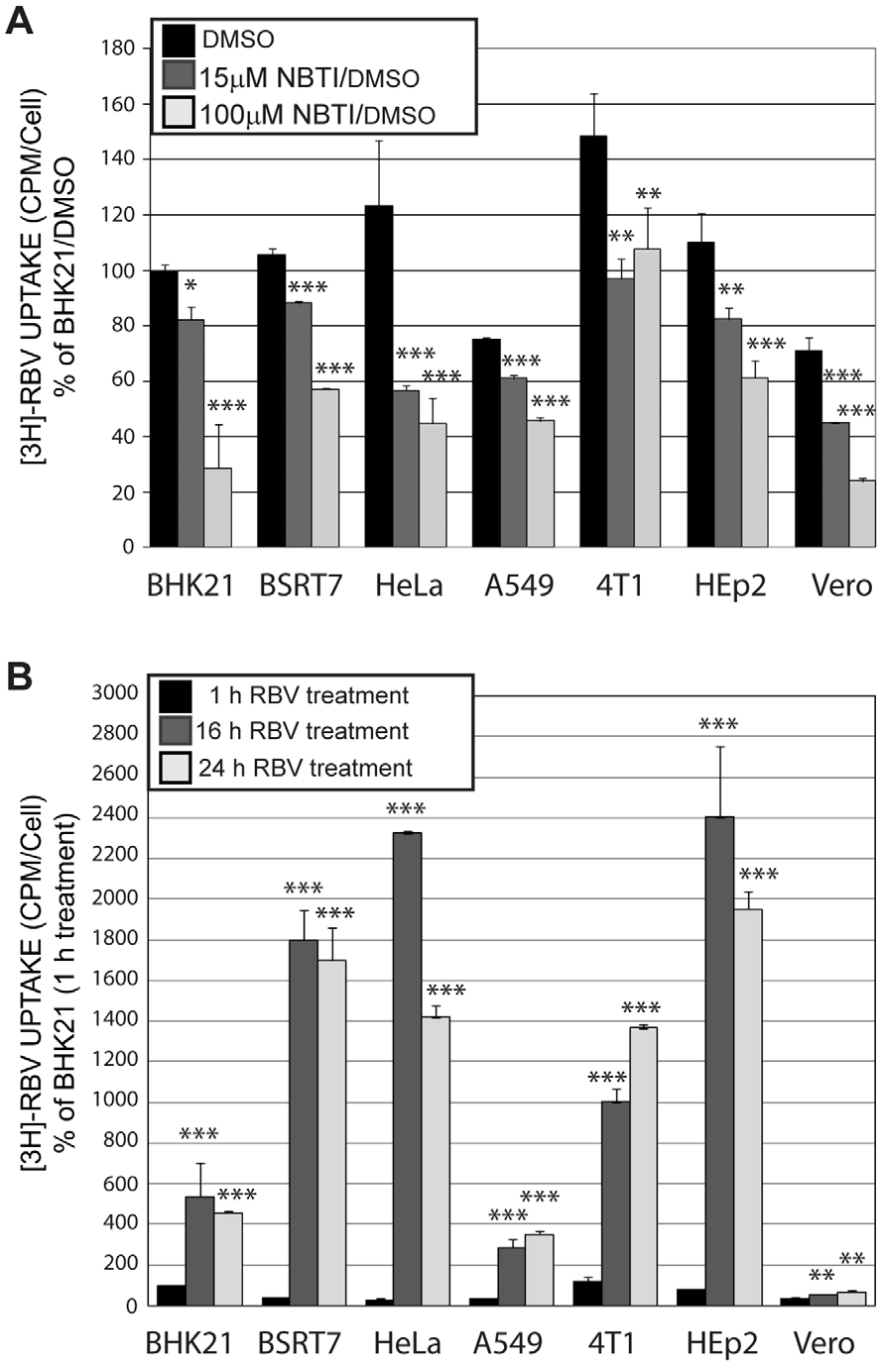
RBV uptake and its inhibition in different cell lines. (**A**) Cell monolayers on 24-well plates (done in triplicates) were pretreated for 15 minutes with 15 or 100 µM NBMPR/DMSO or mock-treated with the same amount of DMSO as contained in the treated wells. Cells were then treated with SFM containing 50 µM RBV 1% of which was [^3^H]RBV for 15 minutes at 37°C. Nucleotide pools were isolated and measured for [^3^H] as described in Materials and Methods. Uptake values represent CPM divided by number of cells in a 24-well plate and normalized to the uptake by DMSO-treated BHK21 cells (defined as 100%). The mean ± standard deviation is shown for four independent experiments (done in triplicates). (B) Cells monolayers (done in triplicates) on 12-well plates were treated with SFM containing 50 µM RBV 1% of which was [^3^H]RBV (without uptake inhibitors) at 37°C for 1 h, 16 h or 24 h. Nucleotide pools were isolated and measured for [^3^H] as described in Materials and Methods. Uptake values represent CPM divided by number of cells in a 12-well plate and normalized to the uptake by BHK21 cells for 1 h (defined as 100%). The mean ± standard deviation is shown for two independent experiments (done in triplicates). (A–B) Statistical analysis was done using one-way ANOVA with Tukey's post hoc test (GraphPad Prism 4, San Diego, CA). ***P<0.001, **P<0.01, *P<0.05, as compared to RBV only treated cells (A) or cells treated with RBV for 1 h (B).

A recent study by Ibarra and Pfeiffer (2009) showed that the development of cell-based resistance to RBV treatment via decreased RBV uptake can greatly limit RBV antiviral activity. Therefore, we wanted to examine a possibility that the RBV resistance of Vero, BHK21 and A549 cells was a result of defective RBV uptake in these cell types, using methodology similar to that described previously [16]. To measure RBV short-term uptake, cells were treated with SFM (same media type used for infections but without virus) containing 50 µM RBV (1% of which was [^3^H]RBV). After 15-minute incubation, cells were collected and measured for the level of [^3^H]RBV uptake normalized to the number of cells as described in Materials and Methods. As shown in Figure 4A (black bars), all tested cell lines showed somewhat similar levels of RBV import after 15-minute incubation, indicating that none of the tested cell lines was defective in RBV uptake. To confirm that the slightly lower [^3^H]RBV counts presented in Figure 4A for BHK21, A549, and Vero cells reflect active uptake of RBV into the cells (rather than background counts), we also analyzed RBV uptake in cells pretreated with increasing concentrations of nitrobenzylthioinosine (NBMPR), a specific inhibitor of equilibrative nucleoside transport via ENT1 (inhibited at lower NBMPR concentrations) and ENT2 (inhibited at higher NBMPR concentrations) nucleoside transporters, which were (especially ENT1) previously shown to be primarily responsible for RBV import into the cells [28], [29]. Our results clearly showed RBV uptake was inhibited in most cell lines at both lower (15 µM) and higher (100 µM) NBMPR concentrations (Fig. 4A), confirming that ENT play at least some role in the influx of RBV into all tested cell types. Interestingly, we were unable to see any additional decrease of RBV uptake in 4T1 cells at the higher NBMPR concentration (100 µM) where both ENT1 and ENT2 are inhibited [16]. However, a decrease was observed at 15 µM NBMPR concentration, suggesting that ENT1 is involved in the RVB uptake in this cell line.

While our short-term uptake experiments did not reveal any defects in RBV import in the seven cell lines, we wanted to see whether long-term accumulation of [^3^H]RBV, which depends on the RBV metabolism) was different in the seven cell lines. To test it, we conducted a similar uptake experiment described above but with cells treated with [^3^H]RBV for 1 h, 16 h and 24h (instead of 15 minutes). As shown in Figure 4B, dramatic variations were observed in the long-term accumulation of RBV in different cell types. Importantly, it correlated with the antiviral efficacy of RBV in the tested cell lines. Thus, all 3 RBV-resistant cell lines, BHK21, A549 and especially Vero showed markedly decreased levels of RBV accumulation suggesting that such the differences in the intracellular RBV metabolism may be responsible for natural resistance of BHK21, A549 and Vero cells to antiviral RBV treatment [16].

### Neutralizing effect of guanosine and actinomycin D addition on the antiviral activity of RBV

**Figure 5:**
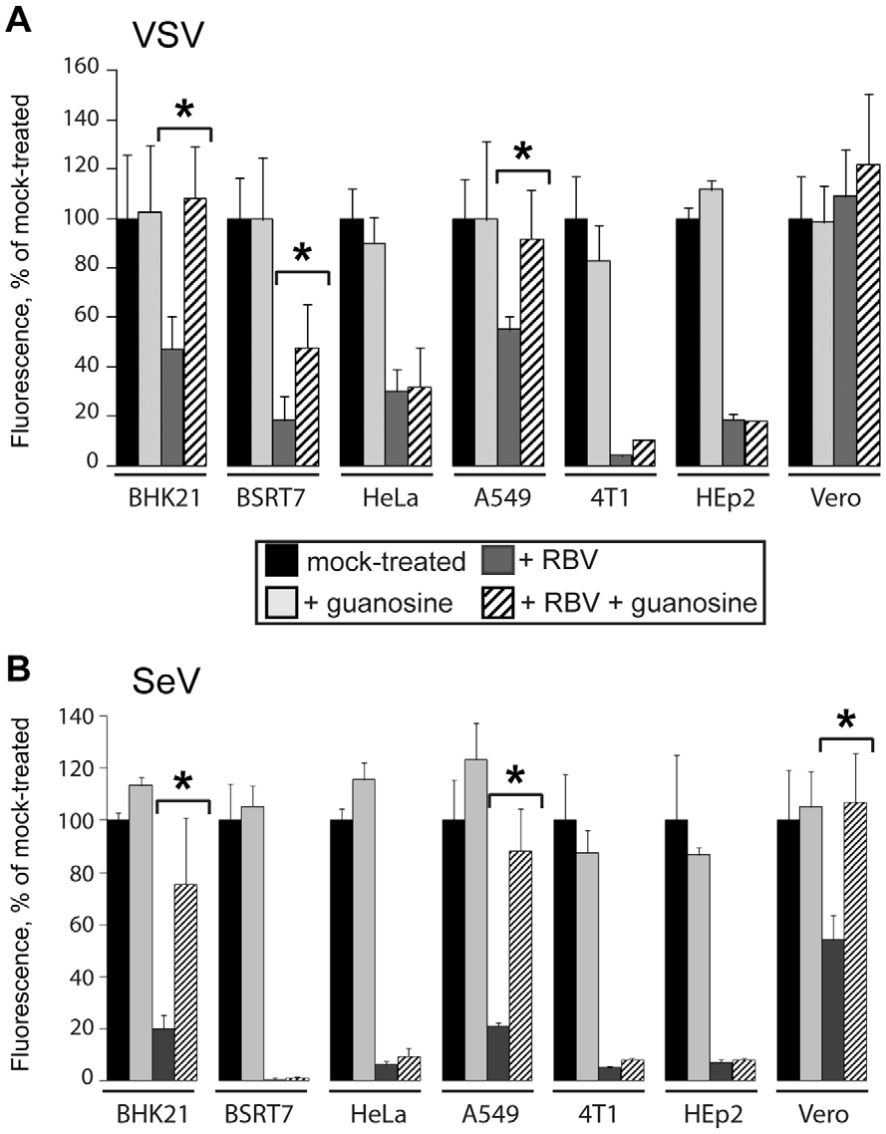
Effect of exogenously added guanosine on antiviral activity of RBV. Cells were mock infected or infected with either VSV-GFP or SeV-GFP at MOI of 3 CIU/cell, and then mock-treated or treated with SFM containing 500 µM RBV, 50 µM guanosine, or both. The intensity of GFP fluorescent signal at 18 h p.i for VSV (A) and 24 h p.i for SeV (B) was quantified using a 96-well plate reader, as described in Materials and Methods. Each of these experiments was performed twice (done in triplicates) and data points represent the mean ± standard deviation. (A–B) Statistical analysis was done using one-way ANOVA with Tukey's post hoc test (GraphPad Prism 4, San Diego, CA). ***P<0.001, **P<0.01, *P<0.05 are shown to compare RBV plus guanosine treatment against RBV treatment only.

One of the major proposed mechanisms of RBV antiviral action is the inhibition of the host enzyme IMPDH essential for the *de novo* synthesis of GTP. Moreover, a recent study suggests that inhibition of IMPDH and the consequent decrease in the cellular GTP pool (but not interactions of RBV metabolites with viral polymerase) is the predominant mechanism of action of RBV against RSV (a paramyxovirus) [30]. To examine whether RBV inhibits VSV and SeV in all seven tested cell lines primarily via depletion of the GTP pool, we analyzed the effect of exogenously added guanosine on the antiviral effect of RBV. If GTP depletion alone is sufficient for inhibition of viral replication, we expected complete neutralization of the RBV effect in cells treated with a combination of RBV (500 µM) and guanosine (50 µM). The selected 50 µM guanosine concentration should result in dramatic increase in the intracellular GTP levels. According to previous studies, even 10 µM exogenous guanosine produces at least 4-fold excess of physiological GTP levels within Vero, HepG2, MDCK and other cell lines [30], [31], [32]. Cells were infected with either VSV-GFP or SeV-GFP at MOI of 3 CIU/cell, and then mock-treated or treated with the SFM containing RBV or guanosine, or RBV together with guanosine. The intensity of GFP-associated fluorescence attributable to viral replication was quantified (as described in Materials and Methods) at 18 h p.i for VSV and 24 h p.i for SeV (Fig. 5). As expected, guanosine treatment alone had no significant effect on virus replication (Fig. 5) in most cell lines. It had also a clear neutralizing effect on RBV in BHK21 and A549 cells, already highly resistant to RBV (Fig. 5). Intriguingly, guanosine had an intermediate neutralizing effect in BSRT7 cells for VSV and a very small effect on RBV activity in the RBV-sensitive HeLa, 4T1 and HEp-2 (and BSRT7 for SeV) cells (Fig. 5), although all tested cell lines had somewhat similar levels of [^3^H]-guanosine uptake (data not shown). The addition of 50 µM guanosine was unable to neutralize the RBV effect in these 4 cell lines even when the RBV concentration was lowered to 200 or 100 µM (data not shown). Also, a similar result was obtained when 200 µM guanosine was added to the medium (data not shown). These data suggest that a decrease in the cellular GTP pool is not the predominant mechanism of RBV action against VSV and SeV in HeLa, 4T1, HEp-2 and BSRT7 cells, and that other mechanisms also contribute to RBV activity against these two viruses in those cell lines.

**Figure 6:**
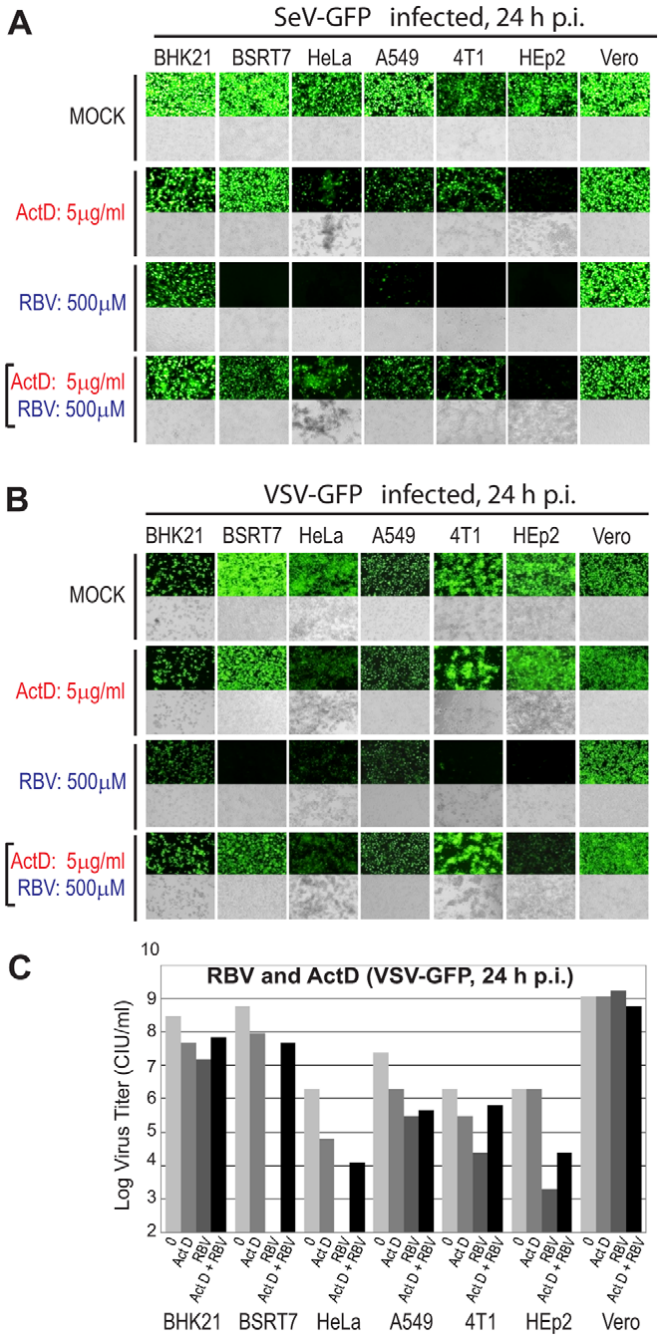
Effect of ActD on antiviral activity of RBV. Cell monolayers were infected with SeV-GFP (A) or VSV-GFP (B) at MOI 3 CIU/cell in the absence of drugs, or with 5µg/ml ActD, 500 µM RBV, or both. Fluorescence (upper panels) and light (lower panels) microscopy images were captured at 10× magnification. The photographs are typical representations of at least three independent experiments and an average field for each well is shown. (C) The number of new infectious VSV-GFP particles generated in the wells photographed in (B) was determined by analysis of SFM collected from each well by plaque assay on BHK21 cells (done in duplicates, average is shown).

Previous studies showed that actinomycin D (ActD), an inhibitor of DNA-primed RNA synthesis (but not viral RNA-dependent RNA synthesis), was able to revert the antiviral effect of RBV against several RNA viruses, including VSV [17], RSV [33], Sindbis virus [34] and rotavirus [35]. Two mechanisms of such reversion were proposed including the stabilization of cellular GTP levels [17], [33], [34], [35] and inhibition of ribavirin triphosphate (RTP) production [33]. To examine whether RBV neutralization by ActD can be also reproduced in case of SeV and whether it is cell type dependent, we infected cells with VSV-GFP or SeV-GFP at MOI 3 CIU/cell and treated these cells with ActD (5 µg/ml) or RBV (500 µM) alone or with both drugs together at 1 h p.i. Photographs of infected cells were taken at 24 h p.i. and the media from each well was collected and titered to determine the number of new infectious particles produced. As shown in Figure 6 (A–C), ActD had a clear neutralizing effect on RBV in most cell lines, while it had a somewhat mild effect on viral replication when used alone in most cell lines with the strongest negative effect observed in HEp2 cells for SeV and HeLa cells for VSV. The tolerance of both viruses to ActD treatment is consistent with a relative independence of their exclusively cytoplasmic replication cycle on new mRNA synthesis by cellular RNA polymerase II, a target of ActD. To rule out a possibility that ActD treatment affected RBV import into the cells, all seven cell lines were treated with ActD (or mock-treated) for 2 h followed by a [^3^H]RBV uptake experiment conducted as described in Materials and Methods. Our results showed that ActD treatment did not inhibit RBV uptake, but actually resulted in a slight increased uptake of RBV (data not shown), demonstrating that the observed reversal of RBV antiviral action (Fig. 6) was not due to the interference of ActD with RBV uptake.

### Resistance of cell lines to RBV and their ability to support viral replication

**Figure 7:**
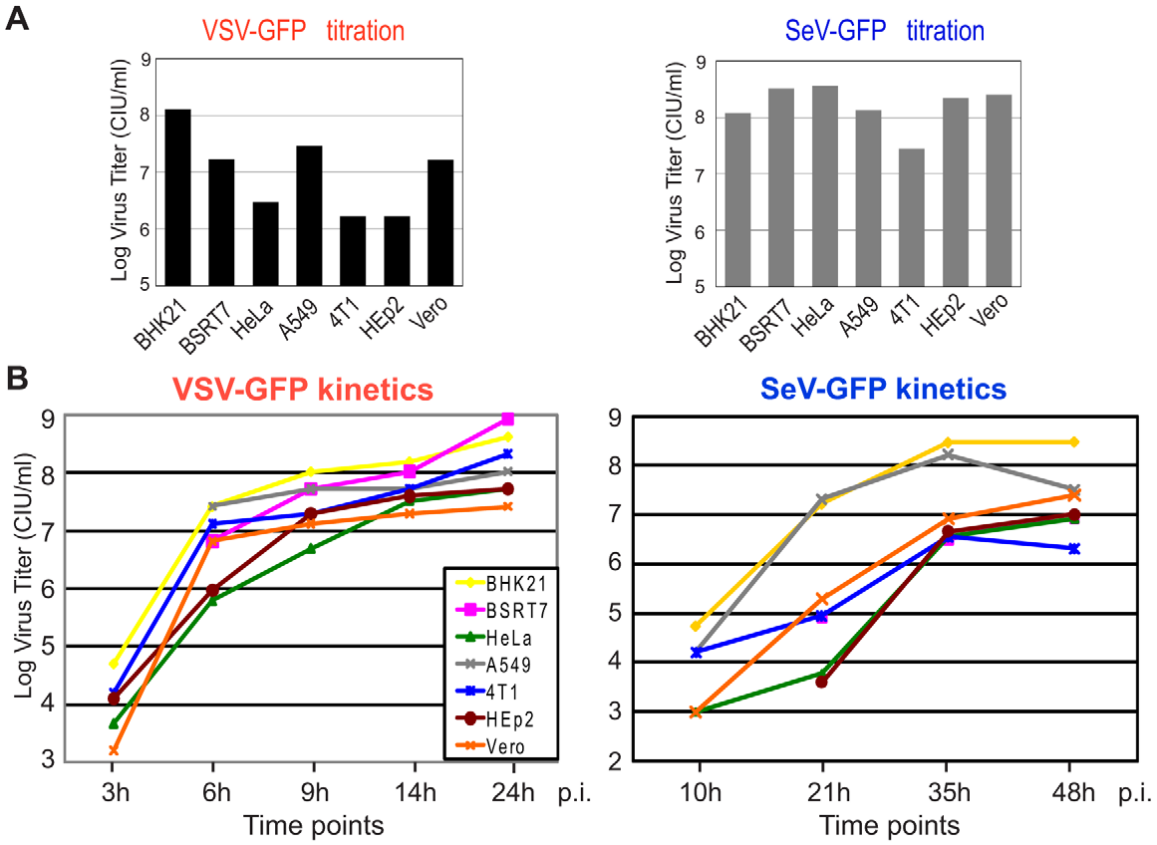
Viral infectivity and replication kinetics in the seven cell lines. (A) Cells were infected with serial dilutions of VSV-GFP (left) or SeV-GFP (right), and infectious foci were counted to calculate the infectivity of the viral stock for each cell line. (B) One-step kinetics of viral replication in seven cell lines. Cells were infected in parallel with VSV-GFP or SeV-GFP at MOI of 3 CIU/cell (1 h absorption), washed 3 times with PBS, and kept in SFM. The media containing newly generated virions was collected at the indicated time points and viral titrations were performed on BHK21 (for VSV) or Vero cells (for SeV).

As noted, the seven cell lines used in this study were selected solely based on their ability to support replication of VSV and SeV. To assess any possible correlation between the general ability of these viruses to replicate in these cell lines and their resistance to RBV, we compared VSV and SeV for their ability to initiate infection and for their replication kinetics in these cell lines without RBV treatment. First, VSV-GFP or SeV-GFP virus stocks were titrated in parallel on different cell lines and the relative ability of each virus to initiate virus infection was calculated by counting infectious foci generated on each cell line. As shown in Figure 7A (and Table 1 with the numbers calculated based on the Figure 7A data), Vero, BHK21 and A549 cells, all highly resistant to RBV, were among the four cell lines most susceptible to VSV infection. Consequently, for our MOI 3 infections described in Figures 1, 3, 5 and 6, to achieve VSV MOI 3 infection for each cell line, for each 3 µl of the VSV-GFP virus stock added to the RBV-resistant BHK21 cells (13.2 µl to A549, 23.7 µl to Vero), 227 µl of the same stock was added to the RBV-sensitive 4T1 and HEp2 and 132 µl to HeLa cells (Table 1). However, RBV-sensitive BSRT7 cell line was found to be as susceptible to VSV as the most RBV-resistant Vero cells (Figure 7A and Table 1). In case of SeV, most cell lines (except for 4T1) showed somewhat similar rates of viral infection initiation for SeV, without any strong correlation with RBV sensitivity (Figure 7A and Table 1).

We also conducted one-step growth kinetics analysis by infecting each cell type with VSV-GFP or SeV-GFP at MOI of 3 CIU/cell (MOI was calculated individually for each virus/cell type combination) and measuring production of new infectious particles by collecting medium from each well at specified time points and titrating it as described in Materials and Methods. While some correlation can be seen in SeV with its fastest growth kinetics (and highest titers) in BHK21, A549 and Vero cells (all three resistant to RBV), it is less apparent in the case of VSV, which grows relatively similarly in most cell lines (Fig. 7B). Together, all these results show no clear correlation between abilities of cell lines to support viral replication and their resistance to RBV, although the abilities of cells to support robust virus replication may be an important factor that would allow successful replication in the presence of RBV as all three RBV-resistant cell lines supported high replication levels of both VSV and SeV. Nevertheless, our results show that virus growth phenotype alone (e.g., VSV in BSRT7) cannot be used to predict efficacy of RBV against VSV or SeV in a given cell line.

## Discussion

In this study, we compared the antiviral activity of RBV against two prototypic members of the order *Mononegavirales*, VSV (a rhabdovirus) and SeV (a paramyxovirus), in seven different cell lines originated from various hosts and tissues. Previous studies showed that RBV can effectively inhibit replication of VSV [17], [18], [19] and SeV [20], [21] as well as other members of *Mononegavirales* [17], [18], [19], [30], [33], [36], [37], [38], [39], [40]. However, in most of these studies only one or two different cell lines were tested. The seven cell lines used in this study were selected solely based on their ability to support replication of both viruses. The two-virus approach allowed us to discriminate between virus specific and cell-based resistance to RBV treatment because, although both viruses belong to the same order *Mononegavirales*, they belong to different families and have noticeably different growth kinetics in these cell lines.

Our results show striking differences between cell lines, ranging from the extremely poor antiviral activity of RBV in Vero cells (e.g., IC_50_ = 2250 µM for VSV and 1550 µM for SeV; IC_90_>3000 µM for both viruses), moderate activity in BHK21 and A549 cells, and very effective inhibition in HEp2, HeLa, 4T1 and BSRT7 cells (IC_50_ = 10 µM, IC_90_ = 40 µM for VSV in BSRT7; IC_50_ = 16 µM, IC_90_ = 40 µM for SeV in BSRT7). This pattern was confirmed using various infection and RBV treatment conditions, with cells infected and treated at 34 or 37°C, high or low MOI, and with RBV treatment starting at 24 h before infection, 6 h before infection, or 1 h p.i. Using three different cell viability assays, we showed that RBV treatment even at 1000 µM concentration did not produce any significant cytotoxicity in any of the tested cell lines at our experimental conditions, nor did we observe any significant differences between tested cell types, indicating that the observed pattern of RBV resistance was not due to differences in RBV toxicity. It is important to emphasize that the median RBV plasma concentration in HCV patients at the peak of RBV therapy is between 6.6and 9 µM [41], [42], [43], [44]. Therefore, the IC_50_ and IC_90_ values for Vero, BHK21 and A549 cells (Table 2) indicate extremely high resistance of these cell types to RBV.

Our data strongly argue that the observed resistance of VSV and SeV to RBV in Vero, BHK21 and A549 was not due to the generation of RBV-resistant mutants in these cells. Such “virus-based” resistance mechanism was previously described for several other RNA virus groups, including polioviruses [5], [12], foot-and-mouth disease virus [13], [14] and recently for HCV [45]. However, even when our cells were treated with RBV starting as early as 24 h before infection (Fig. 1), we observed little effect of RBV on viral replication in RBV-resistant cells, ruling out any possibility of virus adaptation to RBV. In addition, when VSV was passed 10 to 15 times in HeLa, BSRT7 and BHK21 cells in the presence of sub-inhibitory RBV concentrations, no viral adaptation to RBV was ever observed (N.R.S. and V.Z.G., unpublished data). These observations are consistent with a previous study by Cuevas et al. (2005) demonstrating that even after 100 generations under sub-inhibitory concentrations of RBV, resistance of VSV to RBV was not achieved, with selected populations generally less fit than the ancestral population both in the presence and absence of RBV [19].

A recent study by Ibarra and Pfeiffer [16] showed that the development of cell-based drug resistance after continuous RBV treatment via decreased drug uptake can greatly limit RBV efficacy. In addition, any potential antiviral mechanism absolutely relies on RBV entry into the cell. Therefore, we compared our seven cell lines for their ability to internalize RBV. Our results showed a similar RBV uptake in all tested cell lines after 15-minute treatment, indicating that none of the tested cell lines was defective in RBV uptake. In addition, using NBMPR, a specific inhibitor of equilibrative nucleoside transporters, we confirmed that ENT1 and possibly ENT2 transporters are involved in the RBV uptake [29], [46]. A similar RBV uptake level by all tested cell lines is not surprising as ENTs are ubiquitously expressed in virtually all cell types [49]. However, when we analyzed long-term RBV accumulation in cells after 16 h or 24 h treatment, a totally different picture was observed. Four cell lines sensitive to RBV (BSRT7, HeLa, HEp2 and 4T1) showed significantly higher levels of RBV accumulation compared to RBV-resistant BHK21, A549 and Vero. Vero cells had particularly low accumulation which may explain the highest resistance of this cell line to RBV treatment among all the cell lines tested in our study (Table 2).

It is important to note that while the 15-minute uptake assay determines the ability of cells to internalize RBV, the long-term accumulation is dependent on the cellular metabolism of RBV. Neutral RBV molecule can be transported freely in and out of a cell via ENTs, but once it is phosphorylated, negative-charged RMP, RDP, or RTP are trapped inside the cells. A good illustration of the difference between the RBV uptake and its long-term accumulation is RBV hyperaccumulation in erythrocytes resulting in haemolytic anemia in some RBV-treated patients. Similarly to nucleated cells, RBV is transported into erythrocytes via ENTs [46] and converted into RMP, RDP and RTP. However, unlike nucleated cells, they lack the phosphatases needed to hydrolyze RMP/RDP/RTP into RBV [47], [48], [49]. Recent study by Endres et al. (2009) directly showed that total radioactivity of RBV after long-term administration is predominantly attributed to RMP and RTP [49]. Hyperaccumulation of these molecules, along with other factors, results in cellular toxicity of erythrocytes and subsequent anemia [47].

While future studies are warranted to directly analyze RBV metabolism in the seven cell lines, our results indicate that these cell lines may significantly differ in their abilities to accumulate sufficient amounts of phosphorylated RBV metabolites required for effective RBV antiviral actions. RMP is believed to play the major antiviral role as a competitive inhibitor of the enzyme IMPDH essential for the *de novo* synthesis of GTP and is also capable of binding and inhibiting at least some viral polymerases [2], including viral polymerase of VSV [17], [18], [19]. RTP may also play an important role in the inhibition of VSV and SeV replication via interaction with viral polymerase (shown for RTP and VSV [18]), “error catastrophe” or any other mechanisms which involves RTP as a substrate for viral RNA polymerase.

To examine whether RBV inhibits VSV and SeV primarily via depletion of the GTP pool, we treated VSV or SeV infected cells with RBV in the presence of extracellular guanosine which restores normal intracellular GTP level. Guanosine had a clear (almost 100%) neutralizing effect on RBV in BHK21, A549 and Vero cells, which are already highly resistant to RBV. However, very little effect was observed on the RBV activities in RBV-sensitive cells, especially HeLa, 4T1 and HEp-2 cells. Together, these data suggest that a decrease in the cellular GTP pool is not the predominant mechanism of RBV action against VSV and SeV in HeLa, 4T1, HEp-2 and BSRT7 cells, and that other mechanisms also contribute to RBV activity against these two viruses in these cell lines.

Unlike guanosine, ActD was able to effectively neutralize RBV in all tested cell lines. Previous studies showed that ActD neutralizes RBV effects via two mechanisms (likely not mutually exclusive). Malinoski and Stollar (1980) showed that ActD neutralized effect of RBV against Sindbis virus by maintaining the GTP pool size at its normal level (the mechanism of this stabilization is still unknown) [34]. A similar effect of ActD on GTP pool stabilization was shown by Smee and Matthews (1986) in RSV-infected cells treated with RBV. However, they also analyzed the metabolism of RMP to its mono-, di-, and triphosphate derivatives in uninfected and RSV-infected cells, and concluded that ActD also neutralized RBV effect via inhibition of RTP production [33].

Based on the ability of ActD (but not guanosine) to neutralize the effect of RBV in RBV-sensitive cell lines (HeLa, 4T1, HEp-2 and BSRT7), we hypothesize that RBV antiviral activity in these cell lines depends not only on the depletion of the GTP pool (can be restored by guanosine addition) but also on the successful 5′-phosphorylation of RBV into RMP/RDP/RTP [9], [10], [11], which were previously shown to inhibit VSV RNA synthesis in vitro [18]. At the same time, we think that RBV acts in RBV-resistant cell types (BHK, A549 and Vero) primarily via depletion of GTP pool due to insufficient amounts of phosphorylated RBV molecules in these cells, explaining why the effect of RBV can be completely reversed in these cell lines by guanosine. Further experiments are planned to test this hypothesis and further investigate the mechanism of RBV neutralization by ActD. Overall, our data point out to an interesting possibility that the mechanism of virus inhibition by RBV may be more dependent on cell type than we currently expect. This could explain numerous conflicting reports regarding the “true” mechanism of RBV action proposed by different research groups for the same virus [2], [3], [4], [5]. Furthermore, we anticipate that different results for other viruses might be obtained in the cell lines utilized here. For example, a recent study demonstrated an effective inhibition of canine distemper virus (CDV, family *Paramyxoviridae*, genus *Morbillivirus*) in Vero cells [IC_50_ = 20–50 µM, IC_80_ = 40–110 µM] [39]. This result suggests that CDV and SeV might be inhibited by RBV via different mechanisms.

At present, we cannot explain dramatic differences between BHK21 and BSRT7 cells in their resistance to RBV and the long-term RBV accumulation. BSRT7 cell line is derived from BHK21 and constitutively express bacteriophage T7 polymerase under control of the cytomegalovirus promoter and the neomycin resistance gene [22]. Although we cannot explain why these two cell lines are so different in regard to RBV, we also noticed significant differences in cell appearance, cell growth kinetics, viral growth kinetics and the phenotype of infectious foci for VSV and SeV between BHK and BSRT7 cells (data not shown), suggesting that some additional changes were introduced into BSRT7 when or since this recombinant cell line was generated, or that T7 polymerase expression may be responsible for some or all of those phenotypes.

We believe the very similar pattern of RBV activity against VSV and SeV in seven different cells lines may indicate that these two viruses are inhibited by RBV via the same mechanism. Although the mechanism of SeV (genus *Respirovirus*) inhibition by RBV has not been previously studied, a previous study on RSV (another member of the family *Paramyxoviridae*, but belongs to the genus *Pneumovirus*) suggests the predominant mechanism of action of RBV against RSV is inhibition of cellular IMPDH activity by RMP (and consequent decrease in the cellular GTP pool) rather than interactions of RBV metabolites with the viral polymerase [30]. In contrast, a previous study using in vitro transcription reactions with purified VSV virions demonstrated that RMP, RDP and RTP significantly inhibited viral polymerase activity and hypothesized that these molecules reversibly inhibit an initiating step of VSV RNA synthesis [18]. Further experiments are needed to examine molecular mechanisms of VSV and SeV inhibition by RBV.

Overall, our data demonstrate the antiviral activity of RBV is naturally limited in many cell types which may explain at least some RBV treatment failures. Further studies aimed at the understanding molecular determinants responsible for cell-based resistance to RBV are warranted. This understanding may become an important tool for tailoring individualized treatments with RBV (and possibly other nucleoside analogs) against important viral pathogens. Future experiments are also needed to determine whether the observed differences between different cell lines are limited only to nonsegmented negative-strand RNA viruses by analyzing effect of RBV on replication of positive-strand RNA or segmented negative-strand RNA viruses in these cell lines. Finally, our results strongly point out the importance of using multiple cell lines of different origin when antiviral efficacy and potency are examined for new as well as established drugs in vitro.
